# Modulation of the Systemic Immune Response in Suckling Rats by Breast Milk TGF-β2, EGF and FGF21 Supplementation

**DOI:** 10.3390/nu12061888

**Published:** 2020-06-24

**Authors:** Paulina Torres-Castro, Blanca Grases-Pintó, Mar Abril-Gil, Margarida Castell, María J. Rodríguez-Lagunas, Francisco J. Pérez-Cano, Àngels Franch

**Affiliations:** 1 Section of Physiology, Department of Biochemistry and Physiology, Faculty of Pharmacy and Food Science, University of Barcelona, 08028 Barcelona, Spain; mtorreca29@alumnes.ub.edu (P.T.-C.); blancagrases@ub.edu (B.G.-P.); mariadelmar.abril@ub.edu (M.A.-G.); margaridacastell@ub.edu (M.C.); mjrodriguez@ub.edu (M.J.R.-L.); angelsfranch@ub.edu (A.F.); 2 Nutrition and Food Safety Research Institute (INSA·UB), 08921 Santa Coloma de Gramenet, Spain

**Keywords:** growth factors, breast milk, neonatal rats, systemic immunity

## Abstract

Breast milk is a rich fluid containing bioactive compounds such as specific growth factors (GF) that contribute to maturation of the immune system in early life. The aim of this study was to determine whether transforming growth factor-β2 (TGF-β2), epidermal growth factor (EGF) and fibroblast growth factor 21 (FGF21), compounds present in breast milk, could promote systemic immune maturation. For this purpose, newborn Wistar rats were daily supplemented with these GF by oral gavage during the suckling period (21 days of life). At day 14 and 21 of life, plasma for immunoglobulin (Ig) quantification was obtained and spleen lymphocytes were isolated, immunophenotyped and cultured to evaluate their ability to proliferate and release cytokines. The main result was obtained at day 14, when supplementation with EGF increased B cell proportion to reach levels observed at day 21. At the end of the suckling period, all GF increased the plasma levels of IgG1 and IgG2a isotypes, FGF21 balanced the Th1/Th2 cytokine response and both EGF and FGF21 modified splenic lymphocyte composition. These results suggested that the studied milk bioactive factors, mainly EGF and FGF21, may have modulatory roles in the systemic immune responses in early life, although their physiological roles remain to be established.

## 1. Introduction

The World Health Organization (WHO) recommends exclusive breastfeeding during the first months of life [1]. Breastfeeding is the natural way to provide newborns with the nutrients they need for healthy growth and development and plays a major role in infectious disease prevention, with common infections making up less than half of the mortality rate.

Breast milk has a perfect dynamic composition of nutrients and bioactive factors involved in the protection of the baby [2,3,4]. It changes from colostrum, the first fluid produced by mothers after delivery, through to transitional milk, which is produced in the middle lactation period, to mature milk, which lasts until the end of the lactation period [2,5,6]. All three types of breast milk have different characteristics in terms of volume, appearance and composition [7]. Whereas colostrum has the highest concentration of immune factors, transitional milk, although different, shares some of its characteristics with mature milk because it is produced in a critical period to allow the nutritional and functional requirements of the rapidly developing newborn [5,8].

Breast milk has many bioactive factors, such as oligosaccharides, hormones, enzymes, immunoglobulins (Ig), lactoferrin, growth factors (GF), cytokines, anti-inflammatory agents and microorganisms. Although they are not fully understood, they are implicated in a wide range of benefits for the neonate and probably in the prevention of some diseases, such as asthma, atopy, diabetes, obesity and inflammatory bowel disease [9,10,11]. These components of immunity are transferred to the newborn from the mother, providing an ideal environment that protects against infection and helps in the development of the infant’s intestinal mucosa, microbiota and their own immunological defenses [12]. Thus, some of these components are being developed and tested for potential medical applications as prophylactic or therapeutic treatments [5].

In particular, breast milk GF exert diverse effects on the intestinal tract, vasculature, nervous system and endocrine system of babies [5]. Breast milk contains a large number of GF in high concentrations, which change between each lactation period [5,13]. The transforming growth factor (TGF)-β family constitutes the most abundant cytokine of breast milk and consists of three isoforms (TGF-β1, TGF-β2 and TGF-β3) [14,15,16]. Although human breast milk contains all the isoforms, TGF-β2 comprises 95%, with its levels being higher in early milk and reduced toward weaning [17,18]. In humans and mice, TGF-β in breast milk is associated with oral tolerance induction in newborns through IgA antibody production modulation and its participation in the differentiation of regulatory T cells [19,20].

Epidermal growth factor (EGF) is detected in many body fluids, including colostrum and milk [21]. EGF is also one of the most abundant GF in the human milk, more than 500 times higher than other GF [22]; its concentration decreases during lactation, with average levels in colostrum being 2000-fold higher than in mature milk [5]. EGF plays a role in the prevention of necrotizing enterocolitis (NEC), the downregulation of proinflammatory cytokines, the upregulation of anti-inflammatory cytokines and in the maintenance of intestinal barrier function [4,12]. Moreover, it is involved in cellular proliferation and maturation and acts in conjunction with other immune factors to assist in the development of gut-associated lymphoid tissue (GALT) [4].

Finally, fibroblast growth factor 21 (FGF21) is found in human, rat and mouse breast milk, exhibiting levels in humans that do not differ significantly between colostrum and mature milk [23]. In neonates, FGF21 is responsible of the induction of brown adipose tissue (BAT) thermogenesis, which is a key process in the adaptation of neonates to the extrauterine environment [24], and recent findings revealed that FGF21 is involved in controlling neonatal mice digestive and endocrine function in the developing intestinal tract [23].

In a previous study, we demonstrated that supplementation with EGF, TGF-β2 and FGF21 was able to promote intestinal lymphocyte maturation in newborn suckling rats [25]. We hypothesized that they could also possess additional developmental roles at the systemic immune level. In this context, the main objective of this study was to determine whether daily supplementation with these three GF could promote systemic immune development during (day 14) and at the end (day 21) of the suckling period. As pups cannot be separated from their dams during suckling because this would deprive them of breast milk, the supplementation was performed in animals that also received these GF by means of natural breast feeding. Thus, although the physiological roles of these bioactive factors could not be elucidated, any effect on the immune variables analyzed will highlight the targets affected by GF supplementation, therefore suggesting a possible mechanism of action.

## 2. Materials and Methods

### 2.1. Animals

Sixteen G15 pregnant Wistar rats (RjHan:WI, Janvier Labs, Le Genest-Saint-Isle, France) were individually housed in cages under controlled conditions of temperature and humidity in a 12:12 h light:dark cycle until they delivered naturally in the animal facilities of the Faculty of Pharmacy and Food Science. They were fed a commercial diet corresponding to the American Institute of Nutrition 93M formulation [26] (Harlan Teklad, Madison, WI, USA) and water *ad libitum*. The studies were performed in accordance with the Institutional Guidelines for the Care and Use of Laboratory Animals and were approved by the Ethical Committee for Animal Experimentation (CEEA) of the University of Barcelona (UB) and by the Catalonia Government (CEEA-UB Ref. 220/15, DAAM 8521).

### 2.2. Experimental Design

The experimental design and sample size was in line with previous studies [25,27]. With regard to sample size estimation, because of the variability among the dams, three litters were required for each group. This calculation was made by the Appraising Project Office’s program from the Universidad Miguel Hernández de Elche (Alicante), which was used to provide statistically significant differences among groups, assuming no dropout rate and considering a type I error of 0.05 (two-sided). The day after birth was registered as day 1 of life, and litters were unified to nine pups per lactating mother. Each dam and their litter were randomly distributed into four experimental groups according their supplementation (3 dams/group), namely, the reference group (REF), the transforming growth factor-β2 group (TGF-β2), the epidermal growth factor group (EGF) and the fibroblast growth factor group (FGF21) (*n* = 27 pups/group), in order to investigate the effects of these GF on the systemic immune response.

Daily, during the suckling period (from 1 to 21 days of life), all pups were identified, weighed and supplemented with oral gavage with a volume of 10 mL/kg/day of the GF of interest. The pups were separated from their mothers 30 min before oral administration to allow gastric emptying. Handling was done at the same period of the day to avoid modifications in biological rhythms. All these actions were performed as described in previous studies [27,28].

### 2.3. Dietary Supplementation

TGF-β2, EGF and FGF21 groups were supplemented with recombinant human TGF-β2 (97.3% homology with rat), recombinant rat EGF and recombinant human FGF21 (92% homology with rat) (all from Peprotech, Rocky Hill, NJ, USA). These products were reconstituted in phosphate-buffered solution (PBS, pH 7.2) with 0.1% bovine serum albumin (BSA, SeraCare Life Sciences, Milford, MA, USA), according to the manufacturer’s recommendations. The dose of TGF-β2 was 35 μg/kg/day, which was based on the amount of TGF-β2 found in mid-lactation rat milk and milk intake by pups at 4–14 days of age [13]. The dose of EGF was 100 μg/kg/day, which was demonstrated to be effective as a treatment in a rat model of NEC [29]. Finally, the dose of FGF21 was 5 μg/kg/day, an amount that was established in relation to TGF-β2, which was found in a 1:10 ratio FGF21:TGF-β2 [23,30]. The REF group received a matched volume of the vehicle (1% BSA in PBS) used to dilute the GF.

### 2.4. Sample Collection and Processing

Samples were collected at two different end points, with 3 animals from each litter sacrificed on both days, i.e., 14 and 21 of life (total number of animals per sampling day, *n* = 9, 3 for each of the 3 dams in the same group), corresponding to the middle and the end of the rat suckling period, respectively. On these days, pups were anesthetized intramuscularly with ketamine (90 mg/kg, Imalgene, Merial, Spain) and xylazine hydrochloride (10 mg/kg, Rompun, Bayer, Spain) and sacrificed by exsanguination. Blood samples were collected by cardiac puncture and then centrifuged (10,000× *g*, 5 min, and room temperature). After plasma separation, samples were stored at −20 °C until Ig analysis. Subsequently, the thymus, spleen and liver were obtained from each rat and weighed. Data were expressed as relative weight (%, organ weight (g)/ 100 g of body weight). The spleen was also collected and stored on ice until processing.

### 2.5. Immunoglobulin Quantification

Plasma samples were diluted at 1:10,000 for IgA, IgM and IgG isotypes (IgG1, IgG2a, IgG2b and IgG2c) quantification using a commercially available kit (ProcartaPlex Rat Antibody Isotyping Panel, Affymetrix eBioscience, San Diego, CA, USA), according to the manufacturer’s instructions, as in previous studies [31]. Mean fluorescence intensities for each analyte in each sample were detected using the MAGPIX instrument (Luminex Corp., Austin, TX, USA), and the results were analyzed using xPONENT software (Luminex Corp.) in the Flow Cytometry Unit of the Scientific and Technological Centers of the University of Barcelona (CCiT-UB). Data were expressed as µg/mL. Total IgG was calculated adding up all the isotypes. The quantitative determinations were performed with the following limits of detection: IgG1, 1289–940,000 pg/mL; IgG2a, 2332−566,667 pg/mL; IgG2b, 3155–2,300,000 pg/mL; IgG2c, 4801−3,500,000 pg/mL; IgA, 576−420,000 pg/mL; IgM, 233−170,000 pg/mL.

### 2.6. Lymphocyte Isolation from Spleen

Spleens were obtained, weighed and placed in complete culture medium containing Roswell Park Memorial Institute (RPMI 1640, Sigma-Aldrich, St. Louis, MO, USA) with 10% fetal bovine serum (FBS), 1% L-glutamine, 1% penicillin–streptomycin (PenStrep) and 0.05 mM 2-mercaptoethanol, all from Merck (Darmstadt, Germany). Then, the spleens were mashed in sterile conditions through a nylon mesh cell-strainer (40 μm, Falcon Cell Strainers, Corning Incorporated, Corning, NY, USA), and spleen lymphocyte suspensions were obtained after lysis of erythrocytes [32]. Then, cells were centrifuged (500× *g*, 10 min, 4 °C) and resuspended with complete RPMI medium. Cell counting and viability were determined by an automated cell counter after staining dead cells with trypan blue (CountessTM, Invitrogen, Madrid, Spain), following usual laboratory procedures [33].

Lymphocytes were immediately used to analyze phenotypes and ability to proliferate and secrete cytokines after mitogen stimulation.

### 2.7. Spleen Cells Stimulation and Proliferation Assay

Spleen cells (5 × 10^5^ cells/mL) were plated in sterile conditions in 96–well tissue culture plates (TPP^®^, Trasadingen, Switzerland) pre-incubated (2 h, 37 °C and 5% CO_2_) with 200 μL/well of monoclonal antibodies (mAb) anti-CD3 (10 µg/mL) and anti-CD28 (2 µg/mL), both from BD Biosciences (San Diego, CA, USA). Eight wells were used for each sample (4 preincubated with the mitogenic mAb (stimulated cells, SC) and 4 without the stimuli (non-stimulated cells, NSC). Cells were incubated for 48 h at 37 °C and 5% CO_2_. Four hours before the end time of the incubation, 5-bromo-2’-deoxyuridine (BrdU, 20 μL/well, Merck) was added in order to measure its incorporation as an indicator of DNA synthesis in a colorimetric immunoassay [25,27].

Afterwards, the culture plates were centrifuged (210× *g*, 5 min) and supernatants were collected and stored at −80 °C until cytokine analysis. After fixation, the anti-BrdU mAb and peroxidase conjugated anti-BrdU mAb and 3,3,5,5’-tetramethylbenzidine (TMB) were added, following the manufacturer’s recommendations of the BrdU Cell Proliferation Assay Kit (Merck). Color reaction was measured at a 450 nm wavelength in a spectrometer (Multiskan MS, Labsystems, Finland). Data were expressed as proliferation rate (%), which was being = (A/B) × 100, where, A represents ((Abs-SC–Abs-NSC)/Abs-NSC) for the supplemented group and B represents ((Abs-SC–Abs-NSC)/Abs-NSC) for the REF group.

### 2.8. Quantification of Cytokine Secretion

Supernatants obtained after cell activation were used for quantification of IL-2, IL-4, IL-10, IFN-γ and TNF-α concentrations using a ProcartaPlex Multiplex Immunoassay, according to the manufacturer’s instructions (eBioscience) and as described above. The quantitative determinations were performed with the following limits of detection: 2.10−8600 pg/mL, 0.85−3500 pg/mL, 14−55,700 pg/mL, 4.35−17,800 pg/mL and 3.08−12,600 pg/mL for each cytokine, respectively.

### 2.9. Immunofluorescence Staining and Flow Cytometry Analysis

Lymphocytes from spleens were immunophenotyped by immunofluorescence staining and flow cytometry analysis. The spleen lymphocytes (5 × 10^5^ cells/mL) were stained with anti-rat mAb conjugated to fluorescein isothiocyanate (FITC), phycoerythrin (PE), peridinin–chlorophyll-a protein (PercP), allophycocyanin (APC) or APC-cyanine (Cy)7, as in previous studies [25,27]. The mAb used in this study were anti-CD4, anti-CD8α, anti-CD8β, anti-TCRαβ, anti-TCRγδ, anti-NKR-P1A, anti-CD45RA and anti-CD25, all from BD Biosciences, anti-CD103 (αE-integrin) and anti-CD62L from Biolegend (San Diego, CA, USA) and anti-Foxp3 from eBioscience.

Briefly, lymphocyte cells were incubated with a mixture of 10 μL of saturating concentrations of each mouse anti-rat mAb in PBS pH 7.2 containing 2% FBS (Sigma Aldrich) and 0.1% sodium azide (Na3N, Merck) at 4 °C in darkness for 20 min. As Treg were also evaluated as CD4^+^ CD25^+^ Foxp3^+^, extracellular staining with anti-CD4-PE and anti-CD25-FITC mAb was combined with intracellular staining with anti-Foxp3-APC mAb using a fixation/permeabilization specific buffer kit (eBioscience). A negative control staining using an isotype-matched mAb was included in each cell sample. Once the samples were washed, all stained cells were fixed with 0.5% p-formaldehyde (Panreac, Barcelona, Spain) and stored at 4 °C in darkness until analysis by flow cytometry. Analyses were performed in a Gallios^TM^ Cytometer (Beckman Coulter, Miami, FL, USA) in the Flow Cytometry Unit of the CCiT-UB. The obtained data were assessed with FlowJo version 10 software (Tree Star Inc., Ashland, OR, USA). Results are expressed as percentages of positive cells in the lymphocyte population selected according to their forward- and side-scatter characteristics (FSC/SSC) or in a particular selected subpopulation.

For cell subset differentiation, five different mAb panels were used: Panel 1: TCRαβ/NK/CD8α; Panel 2: CD8α/CD8β/TCRγδ; Panel 3: CD103/CD62L/CD8α/CD4; Panel 4: CD45RA/CD8α/CD4/CD25; Panel 5: CD25/CD4/Foxp3. With the first panel, NK cells (NKR-P1A^+^ TCRαβ^-^) and NKT cells (NKR-P1A^+^ TCRαβ^+^) could be differentiated, as well as T TCRαβ^+^ cells (TCRαβ^+^ NKR-P1A^−^) which, in combination with the TCRγδ^+^ cells (obtained using Panel 2), constituted T cell total. B cells (CR45RA^+^) were identified using Panel 4.

### 2.10. Statistical Analysis

All variables in all animals were detected, avoiding the non-detect situation. All statistical analyses were performed using the IBM Social Sciences Software Program (SPSS, version 22.0, Chicago, IL, USA). Levene’s and Shapiro−Wilk tests were applied to assess variance equality and normal distribution, respectively. When the results demonstrated equality of variance and normal distribution, a one-way ANOVA test followed by the Bonferroni post hoc test were performed. Non-parametric tests were performed when normal distribution and equality of variance did not exist. Specifically, Kruskal–Wallis and Mann–Whitney *U* tests were used to assess significance for independent samples. Significant differences were established at *p* < 0.05. All data in the text, tables and figures are expressed as the means ± standard error of the mean (SEM).

## 3. Results

### 3.1. Animal and Organ Weights

The mean body weight of all suckling rats at 14 and 21 days of life was 34.81 ± 0.63 g and 60.09 ± 0.92 g, respectively, without differences between the groups. Likewise, the relative weights of organs (spleen, thymus and liver) did not differ between the REF group and the supplemented groups (Table S1).

### 3.2. Plasmatic IgG, IgM and IgA Concentrations

**Figure 1:**
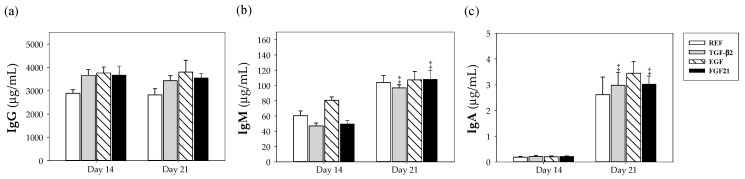
Effect of growth factor supplementation on plasma (**a**) IgG, (**b**) IgM and (**c**) IgA from the four groups, i.e., reference (REF), transforming growth factor-β2 (TGF-β2), epidermal growth factor (EGF) and fibroblast growth factor 21 (FGF21), over the suckling period (day 14 and 21). Results are expressed as means ± S.E.M. (*n* = 9 pups per group). Statistical differences: ‡ *p* < 0.05 vs. same group at day 14.

All studied groups demonstrated increased IgA concentrations (*p* < 0.05 vs. same group at day 14) (Figure 1c) in an age-dependent manner, but this pattern was not found for IgM or IgG. The supplementation of rats with TGF-β2, EGF and FGF21 showed a tendency to increase (~26%) IgG synthesis at both 14 and 21 days with respect to the REF group, without significant differences (Figure 1a). No significant differences were found in plasma concentrations of IgM or IgA between the supplemented groups compared to the REF group (Figure 1b,c). 

### 3.3. Plasmatic IgG Isotypes Concentration

**Figure 2:**
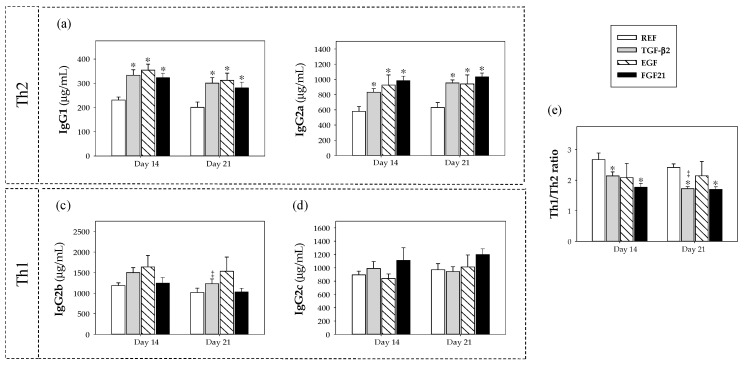
Effect of growth factor supplementation on plasma IgG isotypes from the four groups, i.e., reference (REF), transforming growth factor-β2 (TGF-β2), epidermal growth factor (EGF) and fibroblast growth factor 21 (FGF21), over the suckling period (day 14 and 21). (**a**) IgG1, (**b**) IgG2a, (**c**) IgG2b, (**d**) IgG2c and (**e**) Th1/Th2 antibody pattern ratio. Values (µg/mL) are represented as means ± S.E.M. (*n* = 9 pups per group). Statistical differences: * *p* < 0.05 vs. REF group, ‡ *p* < 0.05 vs. same group at day 14.

In addition to total levels of IgG, the plasma concentrations of each IgG isotype and the Th1/Th2 immune-balance were also studied (Figure 2). Nutritional supplementation with TGF-β2, EGF and FGF21 increased IgG1 (Figure 2a) and IgG2a (Figure 2b) production (Th2-related antibodies in rats [34,35]) without affecting IgG2b (Figure 2c) and IgG2c (Figure 2d) synthesis (Th1-related antibodies in rats [34,35]), with respect to the REF group (*p* < 0.05).

All supplemented groups decreased the Th1/Th2 antibody pattern ratio at day 14 with different percentages of reduction, specifically, TGF-β2 (20%), EGF (22%) and FGF21 (34%). This effect was also found at day 21, when the supplemented groups showed reduced Th1/Th2 ratios by about 29% (TGF-β2), 14% (EGF) and 29% (FGF21). However, only supplementation with TGF-β2 and FGF21 caused a significant decrease in the Th1/Th2 ratio at both days compared to the REF group (*p* < 0.05) (Figure 2e).

### 3.4. Proliferation and Cytokine Production by Spleen Lymphocytes

**Figure 3:**
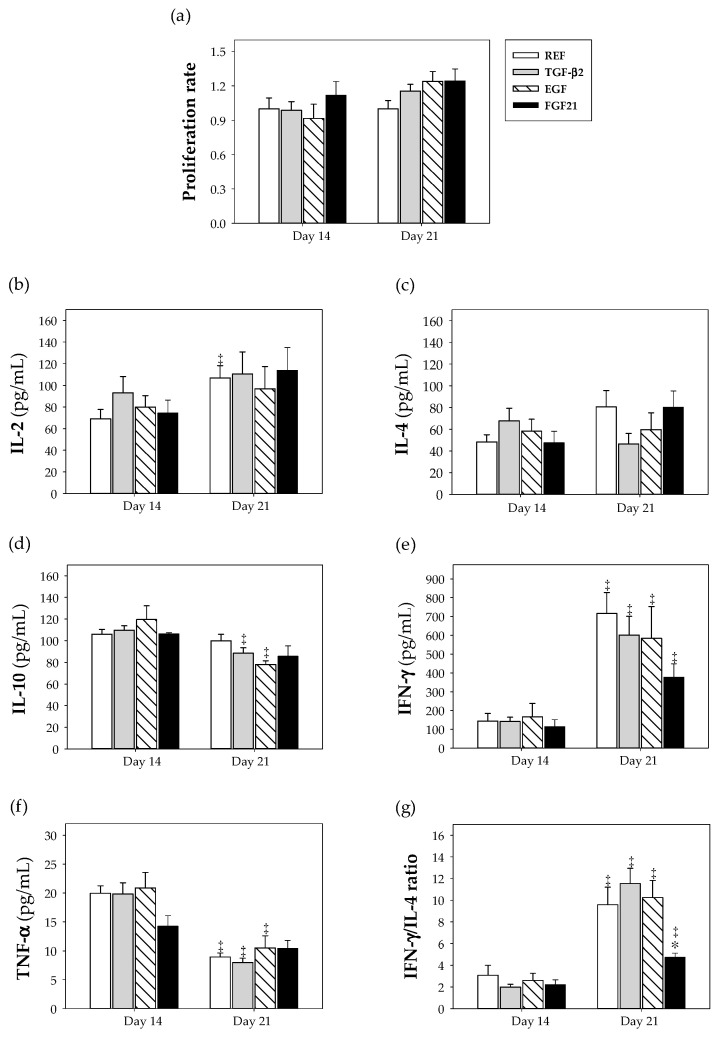
Effect of growth factor supplementation on proliferative response and cytokine production by spleen lymphocytes after in vitro mitogen-stimulation from the four groups, i.e., reference (REF), transforming growth factor-β2 (TGF-β2), epidermal growth factor (EGF) and fibroblast growth factor 21 (FGF21), over the suckling period (day 14 and 21). (**a**) Proliferation rate and cytokine concentration (pg/mL) of (**b**) IL-2, (**c**) IL-4, (**d**) IL-10, (**e**) IFN-γ, (**f**) TNF-α and (g) the IFN-γ/IL-4 ratio. Values are expressed as means (*n* = 9 pups per group analyzed in quadruplicate or duplicate for proliferation assay or cytokine secretion, respectively) ± S.E.M. Statistical differences: * *p* < 0.05 vs. REF group; ‡ *p* < 0.05 vs. same group at day 14.

To determine the functional capacity of suckling rats’ spleen lymphocytes, we studied their proliferative responses and their ability to secrete cytokines induced by anti-CD3 and anti-CD28 mAb during the suckling period (day 14 and 21) (Figure 3a). Regarding proliferation ability, on day 14, GF supplemented groups showed no significant differences with respect to the REF group. At day 21, all supplemented groups exhibited a tendency to increase the proliferation rate by around 15−24%, but without reaching statistical significance.

Concerning cytokine pattern secreted by splenic lymphocytes at days 14 and 21, supplementation did not induce significant changes compared to the REF group. Only some changes associated with age were observed (*p* < 0.05) (Figure 3b–f). However, after calculating the IFN-γ/IL-4 ratio as an indicator of the Th1/Th2 balance (Figure 3g), it was found that supplementation with FGF21 was able to decrease this ratio at day 21 compared to the REF group (*p* < 0.05).

### 3.5. Spleen Lymphocyte Composition

**Figure 4:**
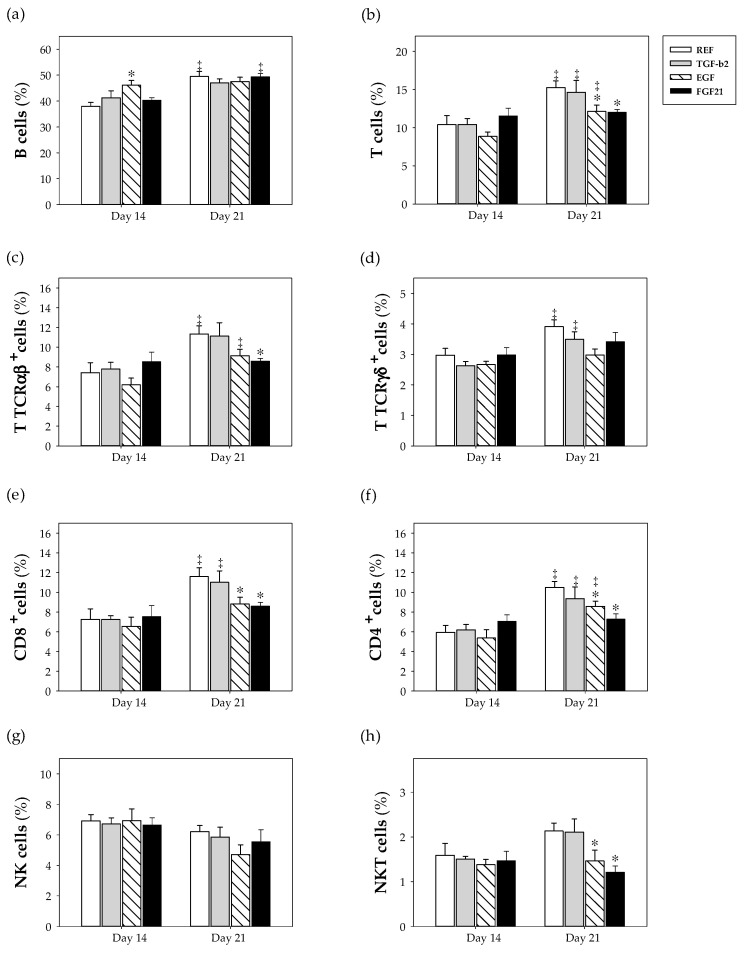
Effect of growth factor supplementation on the main spleen lymphocyte subsets from the four groups, i.e., reference (REF), transforming growth factor-β2 (TGF-β2), epidermal growth factor (EGF) and fibroblast growth factor 21 (FGF21), over the suckling period (day 14 and 21). (**a**) B cells, (**b**) T cells, (**c**) T TCRαβ^+^ cells, (**d**) T TCRγδ^+^ cells, (**e**) CD8^+^ cells, (**f**) CD4^+^ cells, (**g**) NK cells and (**h**) NKT cells. Results (% respect total lymphocytes) are expressed as means ± S.E.M. (*n* = 9 pups per group). Statistical differences: * *p* < 0.05 vs. REF group; ‡ *p* < 0.05 vs. same group at day 14.

The proportions of the main lymphocyte subsets in the spleen were established during (day 14) and at the end (day 21) of the suckling period (Figure 4). In suckling rats’ splenocytes, B cells constituted the main lymphocyte population followed by T TCRαβ^+^ and NK cells and, in smaller proportions, T TCRγδ^+^ and NKT cells (Figure 4).

At day 14, only supplementation with EGF was able to promote the development of B cells (*p* < 0.05 vs. REF group) by increasing its proportion up to levels found in 21-day-old rats (Figure 4a). Regarding day 21, supplementation with EGF and FGF21 did not promote maturation, however, these GF induced a significant decrease in the proportions of the main cell subsets, such as T, CD8^+^, CD4^+^ and NKT lymphocytes (Figure 4b,e,f,h). Moreover, we calculated the B cell/T cell ratio, with all groups showing a value of 4 on both days; only EGF supplementation showed a tendency to increase this ratio (27%) at 14 days of life. In summary, these results showed that EGF and FGF21 supplementation, but not TGF-β2, are able to modify the proportions of the main subsets of spleen lymphocytes. In addition, the Treg cell subsets in all groups at both ages behaved similar, with proportions between 0.5% and 0.7% in all cases.

Further analyses of CD8^+^ and CD8^−^ subpopulations were performed (Table S2). T TCRαβ^+^ and TCRγδ^+^ subsets showed proportions of 29−35% and 60−70% of CD8^+^ cells, respectively, in all groups at both days 14 and 21. Only supplementation with FGF21 decreased (~30%) the proportion of T TCRαβ^+^ CD8^+^ at day 21 of life compared to the REF group (*p* < 0.05). The proportion of CD8^+^ in NKT subset was 78−84% for all groups at 14 and 21 days. The NK subset accounted for 35–40% of CD8^+^ cells at both ages. No significant differences were found in the percentages of NKT and NK subsets in the supplemented groups compared to the REF group.

Expression of the adhesion molecules L-selectin (CD62L) and αE integrin (CD103) displayed no significant differences due to supplementation with respect to the REF group at days 14 and 21 (Table S2). In all cases, the proportion of cells expressing CD62L showed a trend to increase with age (~64% rise, day 21 vs. day 14), and the percentage of cells expressing CD103 tended to decrease with age (~63% of reduction, day 21 vs. day 14), without significant differences observed among the groups.

## 4. Discussion

It is well described that neonatal immune responses are limited, less competent and more functionally deficient than in adults, with newborn rats showing lowers number of immune cells in their lymphoid organs [36]. In this regard, breast milk components have a role in the development of this immature immune system [8].

Some authors researched the effects of TGF-β2, EGF and FGF21 -growth factors present in breast milk- on GALT, but scarce evidence exists regarding the effects of these bioactive factors on systemic immunity in newborn rats [37]. Previously, some research performed by our group demonstrated the effect of the supplementation with TGF-β2, EGF and FGF21 on the promotion of intestinal lymphocyte maturation in newborn suckling rats [25]. The present study was designed to determine whether daily supplementation with these three GF could promote systemic immune development during the postnatal period. For this purpose, the spleen was chosen as a representative systemic lymphoid organ to evaluate immune system status.

It is very difficult to study the particular role of interventional factors normally present in breast milk because of their basal effects; it is possible that the exogenous addition of a breast milk component does not lead to an extra effect. Thus, the best approximation to elucidate the role of a particular component would be to feed a group only with a specific infant formula lacking the particular bioactive factor. However, it is known that without the dam, pups cannot develop appropriately, because they require their mother’s presence not only for nutritional functions, but also for physiological functions, such as body temperature maintenance and urination [38]. In this sense, studies using this artificial feeding approach are limited just to three days of duration [39,40]. However, a couple of studies where rat pups were fed with formula during the whole suckling period, but the intragastrical and intermittent type of feeding in absence of the mother–pup interaction led to altered conditions that did not match the natural physiology [41,42]. Thus, further studies using alternative approaches to avoid the basal value of these GF already present in the breast milk of dams are required. In addition, the use of both physiological doses and higher doses deserve to be explored in the future to better discern the intrinsic effects of each factor.

Some of the bioactive compounds present in breast milk have growth-promoting activities, for example, breast milk EGF acts specifically on the gastrointestinal tract [11,43]. In our study, even though the pups were supplemented with this GF, they also received it from the dams’ breast milk, which may have been the reason for the lack of effect observed on intestinal growth. Moreover, supplementation with TGF-β2 and FGF21 during the whole sucking period also failed to influence the body weight. No changes were detected in the relative weights of spleen, thymus or liver in all supplemented groups, with similar morphometric development observed as in the reference group. In accordance with the present results, previous studies demonstrated that oral administration of EGF (40 mg/kg/day) did not affect liver weight in rabbits [44]. In another study, the thymus and spleen of 4-week-old wild-type and FGF21-knockout mice showed similar weights [45]. However, a strong relationship between breast-fed infants and thymus size was reported in the literature, suggesting the role of breast milk components at this level [46,47]. In light of this, Sakaguchi et al. (2018) demonstrated that the thymus of breast milk-fed pups were slightly larger than that of milk formula-fed mice [48]. Moreover, in the same study, mice from mothers that received anti-TGF-β mAb from delivery until weaning demonstrated a lower average thymus weight than control pups, suggesting that breast milk influences thymus growth and development partly via TGF-β [48]. However, further studies must be conducted, as it is still unclear whether TGF-β directly affects thymic cells. It is well established that both human and rodent newborns have lower antibody concentrations compared to later in life [2,49]. Although no organic alterations were observed, an exhaustive toxicity study of these compounds in this early life stage would be of interest to discard any side effects due to the surplus GF received by the pups.

Our results from plasma IgG, IgM and IgA concentrations in the reference group at days 14 and 21 showed that only IgA increased at the end of the suckling period, in agreement with the findings from other studies [45]. It is known that newborn rodents during the first week of life produce antibodies depending on Th2 responses, in contrast to adult rats, which show a Th1 response [49,50,51]. Thus, the Th1/Th2 balance could indicate the maturation state in young rats; at birth, the immune system is characterized by a predominant Th2 response and maturation is associated with an improved Th1 response [52]. Our results showed increased IgG1 and IgG2a levels in the GF-supplemented groups, which are associated with a Th2 response in rats. These results suggest that all supplementations, especially TGF-β2 and FGF21, may confer immune protection on the pups by favoring the typical response at this stage, the Th2 response. However, and in contrast to our results, it was reported that Brown Norway rats at 18 and 28 days of life that received formula supplemented with TGF-β2 (100 ng/mL) showed a decrease in Th2-associated IgG1 [53]. Differences in the type of feeding (formula vs. breast milk), the age and the strain (because Brown Norway rats are known Th2 responders) may explain this observed differential effect. Thus, further studies should be carried out in order to clarify the role of these GF in humoral immunity development.

Moreover, it is well known that neonatal cytokine secretion is also Th2-oriented, with the Th1/Th2 profile depending on various factors such as nutrition and the microenvironment [54]. It was clearly reported that antigen-presenting cells from the cord blood of human newborns after mitogenic stimulation are prompted to establish a Th2-type cytokine secretion (IL-4, IL-5, and IL-10) pattern over a Th1-type cytokine secretion profile (IFN-γ), thus contributing to Th2 orientation [54,55].

Our results showed that supplementation with TGF-β2 and EGF did not have an overall effect on splenocyte cytokine secretion after mitogen stimulation, and therefore cannot be aligned with the results from the Ig described above and Th1/Th2 shaping. In contrast with the lack of effect in terms of cytokine production of these GF in the current study, it was reported that Brown Norway suckling rats receiving a TGF-β2-supplemented formula between 4 and 18 days of life regulated the Th1/Th2 immune cytokine profile at the mRNA level in the spleen [53].

However, FGF21 supplementation in suckling rats decreased the IFN-γ/IL-4 (Th1/Th2) ratio compared to the REF group at 21 days, thus suggesting a possible enhancement toward the Th2 direction, as also observed in the Ig pattern. It remains to be confirmed if physiological concentrations of FGF21 direct this effect. In fact, very little is known about the immunomodulatory role of this particular GF, which is also considered to be an adipokine, although some authors suggested that FGF21 could be used to counteract inflammatory disorders [56]. One study reported that supplementation with FGF21 in collagen-induced arthritic mice alleviated the disease by down-regulating IL-6, IL-17, IL-1 and TNF-α levels in mouse spleen [57]. In addition, another study with Kunming mice (KM) evaluating supplementation with FGF21 on the toxicity of lipopolysaccharide (LPS) showed that FGF21 significantly decreased the serum contents of inflammatory cytokines, such as IL-1β and TNF-α, whereas it increased those of the cytokine IL-10 [58]. In our study, no effects on inflammatory cytokines were found due to FGF21 administration.

As mentioned in the literature, GF, such as TGF-β2, EGF and FGF21, are compounds with a wide range of biological activities but, from the three studied here, only TGF-β2 and EGF exhibit important roles in cell proliferation and differentiation [12,59,60]. With regard to the capacity of neonatal spleen lymphocytes to proliferate, we observed that regardless of the supplementation of suckling rats, limited lymphoproliferative ability is present during early life. This low spleen lymphoproliferative response during suckling was previously described and suggested to play a key role in the maintenance of tolerance during this period, which may be attributed to lymphocyte immaturity and to the low percentages of functional CD4^+^ and CD8^+^ cells [36]. In contrast with the lack of modulation by GF in our approach, it was shown that Wistar suckling rats receiving oral administration of a whey-enriched TGF-β formula while simultaneously receiving low doses of ovalbumin (OVA) exhibited a down-regulated proliferative-specific splenic cell response [61].

A strength in our approach was the study of the appearance of lymphocyte subsets in the spleen of newborn Wistar rats during the suckling period by means of multiple immunofluorescence and flow cytometry analyses. The lymphocyte composition of rat spleen during suckling shows an immature pattern, which, at weaning, differs from that of adult animals [36]. B lymphocytes constitute the earliest occurring splenic population after birth and, during the suckling period, these cells have an immature phenotype characterized by low surface IgM expression [36]. In the current study, and focusing on this particular subset, EGF supplementation was able to modify spleen lymphocyte composition in suckling rats. At day 14, supplementation with EGF increased the proportion of B cells by ~15%, tended to increase the production of IgM by ~33% and also tended to increase the ratio between B cell and T cell percentages by ~27% with respect to the REF group. The increasing effect of EGF on B cell percentages was also observed in prematurity conditions, confirming its role in the promotion of this cell population [62]. Our results did not show significant differences regarding B cells with either TGF-β2 or FGF21 supplementation. Although very little is known about FGF21 in this regard, it was suggested that TGF-β regulates B cell activation by inhibiting Ig synthesis and class-switching to the majority of IgG isotypes [60]. In line with this, mice from lactating mothers that received anti-TGF-β mAb twice a week from delivery until weaning showed a decrease in B cell proportions [48].

It was described that T cell appearance in neonatal rat spleen is slower than that of B cells, as reflected by the low proportions of T lymphocytes in the first two weeks of life with an increase during the last week of suckling [36], in line with the current results. In addition, other indicators of the stage of immune development in the spleen linked to T cells are the proportions of CD4^+^ and CD8^+^ subsets [36]. Early neonatal life in the spleen (<10 days) is characterized by low proportions of CD4^+^ cells and CD8^+^ cells with immature phenotypes. In the second phase of the suckling period (10–21 days), the numbers of CD4^+^ and CD8^+^ cells increases [36].

Regarding GF supplementation effects at day 21 of life, both EGF and FGF21 decreased the proportions of T cells, CD8^+^ cells and CD4^+^ cells while avoiding the age-related increase observed in the REF group; in contrast, supplementation with TGF-β2 did not alter any of these proportions and this group behaved in the same way as the REF group. Previous studies also described a decrease in T cell percentage after EGF supplementation to preterm rats over 17 days [62]. Regarding FGF21, other studies did not provide comparative information; only a study contradictory to ours was found, which showed that 1- and 4-week-old FGF21-knockout mice exhibited lower percentages of CD4^+^ and CD8^+^ splenic cells, with a thymus-independent action also suggested [45]. These and other results counteracting the age-related evolutionary pattern also suggested an intriguing hypothesis, reflecting the importance of these factors maintaining immaturity rather than inducing it, which could be of importance during this particular period.

NK and NKT cells also constitute a low percentage of spleen lymphocytes during the suckling period. As in previous studies, we found that during the suckling period, spleen NK cells were as abundant as T cells, suggesting that these pivotal cells in innate immunity, play a key role during early life when acquired immune responses are not yet fully developed [36]. Focusing on the effect of the supplementation, none of the GF affected NK proportions, but both EGF and FGF21—but not TGF-β2—decreased the proportion of NKT at day 21. NKT cells are a T cell population that express cell surface markers characteristic of NK cells and T cells; it was suggested that these cells possess an important immune regulatory function by bridging the innate and acquired immune responses [63]. Thus, this modulatory action of EGF and FGF21 could be interpreted as harmful, however, it is possible that it is a way to control the large amounts of Th1 and Th2 cytokines that activated NKT can produce, and may contribute, for example, to the development of IgE-mediated allergic asthma [64,65,66]. Our results indicated that lower proportions of NKT due to EGF and FGF21 supplementation at the end of the suckling period may be beneficial in the prevention of allergic diseases, which are highly prevalent during these stages of life [67,68].

## 5. Conclusions

In summary, although very little information is available with respect to the effect of these growth factors on the systemic immune response in newborns, this study demonstrated that supplementation with TGF-2, EGF and FGF21 have an impact on the immune development. This effect, particularly in the case of FGF21, may be explained in part by the regulation of the Th1/Th2 balance in terms of IgG isotype pattern and associated cytokines that induce Th2 polarization. In some cases, this could also be explained by effects on splenic lymphocyte composition. Further studies are needed to confirm these findings and the differential actions ascribed to each particular breast milk growth factor. Moreover, *ex vivo* or *in vitro* determinations would be also useful to elucidate the mechanism by which they modify the immune system.

## Supplementary Materials

The following are available online at https://www.mdpi.com/2072-6643/12/6/1888/s1, Table S1: Effect of growth factor supplementation on body weight and relative organ weights in suckling rats; Table S2: Effect of growth factor supplementation on the percentages of CD8+ and CD8- lymphocyte subsets and adhesion molecules in spleen lymphocytes of suckling rats.
