# Supplementary material for: Modulation of the Systemic Immune Response in Suckling Rats by Breast Milk TGF-β2, EGF and FGF21 Supplementation
Source: Nutrients. 2020 Jun 24;12(6):1888. doi: 10.3390/nu12061888 (PMC7353385; doi:10.3390/nu12061888)
Supplement: Supplementary file 1 [file nutrients-12-01888-s001.pdf]

nutrients

Supplementary Materials

Table S1. Effect of the supplementation with growth factors on body weight and relative organ weights in suckling rats.

	Day 14							
**Weight**	**REF**		**TGF-β2**		**EGF**		**FGF21**	
	Mean	SEM	Mean	SEM	Mean	SEM	Mean	SEM
Animals (g)	35.53	0.94	35.61	1.56	32.30	0.85	34.62	0.41
Spleen (%)	0.67	0.06	0.70	0.04	0.66	0.03	0.62	0.06
Thymus (%)	0.35	0.01	0.37	0.02	0.36	0.03	0.42	0.05
Liver (%)	3.57	0.11	3.36	0.13	3.52	0.12	3.24	0.15
**Day 21**								
Animals (g)	61.90‡	2.32	60.42‡	1.64	58.40‡	1.37	58.42‡	1.35
Spleen (%)	0.58	0.02	0.61	0.03	0.60	0.03	0.63	0.02
Thymus (%)	0.44‡	0.02	0.45‡	0.02	0.44‡	0.03	0.45	0.02
Liver (%)	4.30‡	0.16	4.52‡	0.14	4.25‡	0.19	4.54‡	0.10

‡ $p<0.05$ *vs.* same group at day 14.

Table S2. Effect of the supplementation with growth factors on the percentage of CD8$^+$ and CD8$^-$ lymphocyte subsets and of adhesion molecules in spleen lymphocytes of suckling rats.

	**REF**		**TGF-β2**		**EGF**		**FGF21**	
**Day 14**	Mean	SEM	Mean	SEM	Mean	SEM	Mean	SEM
**T cells (%)**								
**T TCRαβ$^+$ CD8$^+$/Total**	2.43	0.37	2.47	0.23	1.82	0.22	2.47	0.28
**T TCRαβ$^+$ CD8$^-$/Total**	5.00	0.66	5.32	0.45	4.37	0.48	6.07	0.71
**T TCRαβ$^+$ CD8$^+$/T TCRαβ$^+$**	32.21	1.52	31.64	0.73	29.30	1.01	29.05	1.06
**T TCRαβ$^+$ CD8$^-$/T TCRαβ$^+$**	67.79	1.52	68.36	0.73	70.70	1.01	70.95	1.06
**T TCRγδ$^+$ CD8$^+$/T TCRγδ$^+$**	62.05	2.53	61.03	1.83	63.01	2.48	61.87	2.10
**T TCRγδ$^+$ CD8$^-$/T TCRγδ$^+$**	37.95	2.53	38.97	1.83	36.99	2.48	38.13	2.10
**NKT cells (%)**								
**NKT CD8$^+$/NKT**	83.99	3.02	84.51	1.07	79.74	3.21	79.98	1.53
**NKT CD8$^-$/NKT**	16.02	3.02	15.49	1.07	20.26	3.21	20.02	1.53
**NK cells (%)**								
**NK CD8$^+$/NK**	38.64	2.62	39.19	3.43	31.40	4.12	35.83	2.94
**NK CD8$^-$/NK**	61.36	2.62	60.81	3.43	68.60	4.12	64.17	2.94
**Adhesion molecules (%)**								
**CD103$^+$/Total**	1.82	0.33	1.73	0.22	1.32	0.27	1.34	0.22
**CD62L$^+$/Total**	11.34	1.27	11.95	1.17	10.22	0.87	12.11	0.72
**Day 21**								
**T cells (%)**								
**T TCRαβ$^+$ CD8$^+$/Total**	3.80‡	0.25	3.85‡	0.45	2.94‡	0.27	2.67*	0.10
**T TCRαβ$^+$ CD8$^-$/Total**	7.54‡	0.60	7.28	0.93	6.21‡	0.47	5.93	0.30
**T TCRαβ$^+$ CD8$^+$/T TCRαβ$^+$**	33.77	1.16	34.67	1.40	32.10	1.61	31.20	1.67
**T TCRαβ$^+$ CD8$^-$/T TCRαβ$^+$**	66.23	1.16	65.33	1.40	67.90	1.61	68.80	1.67
**T TCRγδ$^+$ CD8$^+$/T TCRγδ$^+$**	71.83‡	1.84	65.80	3.90	65.30	2.88	65.71	4.09
**T TCRγδ$^+$ CD8$^-$/T TCRγδ$^+$**	28.17‡	1.85	34.20	3.90	34.70	2.88	34.29	4.09
**NKT cells (%)**								
**NKT CD8$^+$/NKT**	79.34	3.08	84.98	1.94	81.95	2.69	78.42	2.89
**NKT CD8$^-$/NKT**	20.65	3.08	15.03	1.94	18.05	2.69	21.58	2.89
**NK cells (%)**								
**NK CD8$^+$/NK**	43.94	1.95	41.03	1.21	41.50	1.78	40.17	1.52
**NK CD8$^-$/NK**	56.06	1.95	58.98	1.21	58.50	1.78‡	59.83	1.52
**Adhesion molecules (%)**								
**CD103$^+$/Total**	0.67‡	0.1	1.35	0.32	0.95	0.24	0.92	0.22
**CD62L$^+$/Total**	18.63‡	1.7	17.22	2.92	15.53‡	1.79	14.28	1.55

*p<0·05 *vs*. reference group, ‡ p<0·05 *vs*. same group at day 14.
